# Predicting Post-Disaster Post-Traumatic Stress Disorder Symptom Trajectories: The Role of Pre-Disaster Traumatic Experiences

**DOI:** 10.3390/ijerph21060749

**Published:** 2024-06-08

**Authors:** Sydney T. Johnson, Susan M. Mason, Darin Erickson, Jaime C. Slaughter-Acey, Mary C. Waters

**Affiliations:** 1 Division of Epidemiology and Community Health, University of Minnesota School of Public Health, 1300 S. 2nd Street, Suite 300, Minneapolis, MN 55454, USA; 2 Department of Sociology, Harvard University, 540 William James Hall, 33 Kirkland Street, Cambridge, MA 01238, USA

**Keywords:** post-traumatic stress disorder, natural disasters, Hurricane Katrina

## Abstract

The mental health impact of disasters is substantial, with 30–40% of direct disaster victims developing post-traumatic stress disorder (PTSD). It is not yet clear why some people cope well with disaster-related trauma while others experience chronic dysfunction. Prior research on non-disaster trauma suggests that an individual’s history of traumatic experiences earlier in the life course, prior to the disaster, may be a key factor in explaining variability in psychological responses to disasters. This study evaluated the extent to which pre-disaster trauma predicts PTSD trajectories in a sample of Hurricane Katrina survivors followed for 12 years after the storm. Four PTSD trajectories were identified using latent class growth analysis: Resistant (49.0%), Recovery (29.3%), Delayed-Onset (8.0%), and Chronic–High (13.7%). After adjusting for covariates, pre-Katrina trauma had only a small, positive impact on the probability of long-term, chronic Katrina-specific PTSD, and little effect on the probability of the Resistant and Delayed-Onset trajectories. Higher pre-Katrina trauma exposure moderately decreased the probability of being in the Recovery trajectory, in which Katrina-specific PTSD symptoms are initially high before declining over time. When covariates were added to the model one at a time, the association between pre-Katrina trauma and Chronic–High PTSD was attenuated most by the addition of Katrina-related trauma. Our findings suggest that while pre-disaster trauma exposure does not have a strong direct effect on chronic Katrina-specific PTSD, pre-Katrina trauma may impact PTSD through other factors that affect Katrina-related PTSD, such as by increasing the severity of Katrina-related trauma. These findings have important implications for the development of disaster preparedness strategies to diminish the long-term burden of disaster-related PTSD.

## 1. Introduction

Research suggests that natural disasters are increasing in both frequency and intensity due to climate change [1]. In 2022, approximately 185 million people were affected by natural disasters worldwide [2]. In 2020, for the first time in US history, 10 hurricanes and tropical storms made landfall in the US [3], and wildfires burned more than four million acres in California, doubling the record of two million acres set in 2018 [3]. As natural disasters become more common, it is crucial to understand how these events affect the health of the rapidly growing population of disaster survivors.

Exposure to disasters increases the risk for post-traumatic stress disorder (PTSD), depression, non-specific psychological distress, and anxiety disorders [4]. PTSD is a mental health condition that is triggered by witnessing or experiencing a traumatic event [5]. A wide range of different types of trauma are associated with PTSD symptoms, including physical violence, sexual assault or rape, and natural or human-made disasters [6]. Although estimates of the prevalence of PTSD among disaster survivors vary significantly between studies due to differences in disaster type, degree of exposure, and other important factors, a literature review spanning 40 years of disaster research found that 30–40% of direct disaster victims develop PTSD [7]. PTSD is characterized by intrusive and distressing remembering or re-experiencing of the event, avoidance, hyperarousal or reactivity, and cognition or mood symptoms [8]. PTSD symptoms can cause difficulty in daily functioning, including an individual’s ability to work or maintain close relationships with family members or friends [8]. PTSD has been called a “life sentence” due to its impact on chronic disease, accelerated aging, and premature mortality [9].

Despite the strong influence of disasters on mental health, not everyone who experiences a disaster develops PTSD and many who do ultimately recover [10]. Research on post-disaster mental health has found that disaster survivors generally follow distinct trajectories of disaster-related PTSD symptoms. Four prototypical trajectories of dysfunction have been identified [11,12,13,14,15]. Many people do not experience persistent disaster-related PTSD symptoms [10]; these individuals either experience few or no PTSD symptoms at all in the aftermath of a disaster (termed “resilience”), or initially experience high PTSD symptoms, but these symptoms decline over time (termed “recovery”) [12]. These two trajectories do not involve persistent PTSD symptoms. However, others experience a chronic or long-term burden of disaster-related PTSD [10]. For example, some disaster survivors initially experience few PTSD symptoms, but these symptoms subsequently increase over time (termed “delayed”) [14]. Finally, some disaster survivors have moderate to severe symptoms that persist over time, resulting in chronic dysfunction (termed “chronic”) [14].

It is not yet clear why some people cope well with trauma while others experience chronic dysfunction. Notably, the severity of disaster-related trauma appears to be only one of many factors that influence mental health in the aftermath of a disaster [11]. Given the variability in psychological responses to natural disasters, understanding who is likely to experience long-term disaster-related PTSD is crucial for allocating limited disaster recovery resources and informing interventions to build resilience.

Much of the research on the long-term mental health effects of disasters has concentrated on experiences during and immediately after a disaster, overlooking potentially important pre-disaster factors that may predict long-term disaster-related mental health problems. Some pre-disaster factors, such as psychological distress, low social support, and limited economic resources, have been identified as risk factors for greater post-disaster PTSD [4]. In addition, low household income, racial/ethnic minority status, and female gender are associated with increased vulnerability to adverse mental health outcomes following a disaster [4,11]. Exposure to traumatic events earlier in the life course prior to a disaster may also help explain variability in psychological responses to disasters.

The broader literature on trauma exposure indicates that having a history of exposure to one or more traumatic events can exacerbate the mental health consequences of subsequent traumatic experiences [16]. The theory of stress sensitization suggests that the stress response systems of individuals exposed to early stressors are primed to respond to later stressors in ways that increase the risk of developing adverse mental health outcomes [17,18]. Repeated traumas have a cumulative effect on health, with increased trauma affecting mental health in a dose–response manner [19], wherein exposure to multiple traumatic events is associated with greater morbidity and impairment in individuals with PTSD [20,21]. As a result, disaster survivors previously exposed to non-disaster-related traumatic events likely experience an excess risk of disaster-related psychological distress and functional impairment, the burden of which can persist for years [19]. Because cases of PTSD in individuals exposed to multiple traumatic events are associated with greater impairment [19], studying the potential impact of pre-disaster trauma is particularly crucial to understand who is most at risk for disaster-related PTSD, and to mitigate long-term psychological distress among social groups disproportionately affected by trauma [20,22].

We hypothesize that experiences of pre-Katrina trauma are associated with an increased risk of chronic Katrina-specific PTSD among survivors. Thus, this study aimed to evaluate the extent to which pre-Katrina traumatic experiences explain differences in long-term trajectories of Katrina-specific PTSD among survivors with similar levels of Katrina-related trauma. As such, the study objectives were to (1) identify PTSD trajectory groups based on patterns of Katrina-specific PTSD symptoms over time; (2) determine the extent to which pre-Katrina trauma exposure predicts the trajectory of Katrina-specific PTSD symptoms an individual will follow and (3) examine whether this association operates independently of the severity of Katrina-related trauma, which may itself be influenced by pre-Katrina trauma.

## 2. Materials and Methods

This paper leveraged survey data from the Resilience in Survivors of Katrina (RISK) study, a longitudinal study of low-income, primarily Black, women who lived in New Orleans at the time of Hurricane Katrina [23]. The RISK sample was drawn from the Opening Doors Demonstration, a randomized-design program developed to increase community college graduation rates and academic achievement among low-income adults with children under age 18. Between November 2003 and June 2005, Opening Doors enrolled 1019 low-income parents when they registered for classes at one of two community colleges in the New Orleans area. As part of the community college intervention study, all participants completed a baseline questionnaire about their educational background and goals, employment history, and sociodemographic characteristics. They also completed a short baseline survey about their physical and mental health, attitudes about schooling, and social relationships.

After Hurricane Katrina disrupted data collection for the Opening Doors 12-month follow-up survey in August 2005, the study was redesigned as RISK to examine the consequences of a disaster on the lives of vulnerable individuals and their families. To qualify for Opening Doors, participants needed to be enrolled in community college, be between the ages of 18 and 34, have at least one child under age 18, and earn less than 200% of the poverty line at baseline. In addition to the pre-Katrina Wave 1 data collected in 2003–2005, the RISK study conducted three post-Katrina follow-up surveys one, four, and twelve years after the hurricane: Wave 2 was collected in 2006–2007 (response rate: 70% of original sample; n = 667), Wave 3 was collected in 2009–2010 (response rate: 75% of original sample; n = 702), and Wave 4 was collected in 2016–2018 (response rate: 76% of original sample; n = 715). Each of the follow-up surveys included questions pertaining to experiences during and after Hurricane Katrina, health resources and outcomes, social networks and support, and economic resources. Of the 1019 original participants, 92% were women. Because so few men were enrolled, these 77 men were excluded from these analyses, leaving a sample of 942 women. The current analysis was limited to women who completed at least two of the three post-Katrina PTSD assessments; 728 of the 942 women in the baseline sample (77%) met this criterium.

### 2.1. Measures

#### 2.1.1. Outcome

Katrina-specific PTSD. Katrina-specific PTSD symptoms were assessed at all post-Katrina timepoints (Waves 2–4) using the Impact of Event Scale—Revised (IES-R), a 22-item scale that evaluates subjective distress related to a specific traumatic event, in this case, Hurricane Katrina [24]. Participants were asked to rate the degree to which they were distressed by hurricane-related difficulties in the past 7 days (e.g., “I stayed away from reminders of it,” and “I was jumpy and easily startled”). For each item, respondents indicated whether they were distressed or bothered by this issue “not at all” (0), “a little” (1), “moderately” (2), “quite a bit” (3), or “extremely” (4). A PTSD score ranging from 0 to 4 was constructed as the mean of all items [25]. Higher scores indicate more severe post-traumatic stress symptoms, and scores above 1.5 are indicative of probable PTSD [25]. Cronbach’s alpha (α) was 0.94 at Time 1, 0.95 at Time 2, and 0.99 at Time 3.

#### 2.1.2. Primary Exposure

Pre- and post-Katrina traumatic experiences. Pre-Katrina traumas were the primary exposure of interest. Post-disaster trauma was included in the models as an adjustment variable because it may exacerbate Katrina-related PTSD [19]. Pre-and post-disaster traumatic experiences were measured retrospectively in the third post-Katrina survey (Wave 4) using an adapted version of the Life Events Checklist (LEC) [26]. The LEC asks respondents to indicate whether they experienced any of 15 potentially traumatic events. Participants who responded “yes” to an item were asked whether they experienced this event before, after, or both before and after Hurricane Katrina, with the exception that item 15 only asked about natural disasters that occurred after Hurricane Katrina. For each event, two binary variables (yes/no) were created, with one indicating that the participant experienced this event prior to Hurricane Katrina and the other indicating that they experienced it after Hurricane Katrina. Affirmative responses were summed to create indices of pre- and post-disaster trauma exposure. Because assaultive traumas are more consistently associated with PTSD and risk for additional future traumatic experiences than non-assaultive traumas [6,27,28,29,30], the five events related to personal experiences of assaultive violence (e.g., being robbed or mugged; raped or sexually assaulted; or being physically hurt by a parent or caregiver) were summed to create counts of pre- and post-disaster assaultive traumas for separate sub-analyses of assaultive trauma. An index of non-assaultive traumas (e.g., experiencing the sudden unexpected death of someone close to you; experiencing a life-threatening illness) was also created.

#### 2.1.3. Covariates

Pre-Katrina sociodemographics. Sociodemographic covariates were included based on prior research showing their association with levels of trauma exposure in general as well as disaster-related trauma [4]. These covariates are age in years, race, a binary variable indicating whether a participant was married or cohabitating with a partner, and a count of public benefits received (i.e., social security income, unemployment, welfare, and/or food stamps). All four sociodemographic covariates were measured at baseline (Wave 1).

Indicators of Katrina-related trauma and hardship. In line with previous research using this dataset [31,32,33], several measures of Hurricane Katrina-related trauma and hardship were used in these analyses. All of these measures were asked in the first and second post-Katrina follow-up surveys (Waves 2 and 3). First, an eight-item trauma scale based on a survey of Hurricane Katrina survivors [34] asked respondents to answer “yes” or “no” to the following questions about their experiences in the week after Katrina: (1) lacked enough fresh water to drink, (2) lacked enough food to eat, (3) felt one’s life was in danger, (4) lacked necessary medicine, (5) lacked necessary medical care, (6) family member lacked necessary medical care, (7) lacked knowledge of safety of children, or (8) lacked knowledge about safety of other family members. Affirmative responses were summed to create scores ranging from 0 to 8. Second, participants were asked whether a family member or close friend died as a result of Hurricane Katrina. Participants who indicated the loss of a family member or friend were coded as 1 (yes) and those who did not were coded as 0 (no). Third, participants were asked to describe the extent of the damage caused by Hurricane Katrina to the home where they were living when Katrina struck. Answer options included “none” (0), “minimal” (1), “moderate” (2), “substantial” (3), and “enormous” (4). In line with previous studies using this dataset [31,32,33], home damage was dichotomized by collapsing the bottom two (none or minimal, coded as 0) and top three categories (moderate, substantial, or enormous, coded as 1), creating a variable that indicates whether a participant experienced moderate to severe home damage.

Pre-Katrina perceived social support. Pre-Katrina perceived social support was measured at baseline using the Social Provisions Scale [35]. This scale is comprised of eight items, such as “there are people I know will help me if I really need it.” Participants indicated the degree to which they agreed or disagreed with each statement. Answer options were “strongly disagree” (1), “disagree” (2), “agree” (3), and “strongly agree” (4). After reverse coding negatively phrased items, total scores were calculated by taking the mean of all items (α = 0.78). Scores ranged from 1 to 4, with higher scores indicating greater perceived social support.

Pre-Katrina psychological distress. Non-specific psychological distress was measured at baseline using the Kessler-6 (K6) scale, a self-report measure used to assess anxiety and mood disorders [36,37]. This scale is comprised of six items that ask, “in the past 30 days, how often did you feel…”: (1) nervous, (2) hopeless, (3) restless or fidgety, (4) so sad or depressed that nothing could cheer you up, (5) that everything was an effort, and (6) worthless. Answer choices were “none of the time” (0), “a little of the time” (1), “some of the time” (2), “most of the time” (3), and “all of the time” (4). Responses to the six items were summed to construct scores ranging from 0 to 24 (α = 0.77) [37]. Higher scores are indicative of greater psychological distress [36]. Scores of 8–12 indicate probable mild to moderate mental illness, and scores of 13 or higher indicate probable serious mental illness.

### 2.2. Analysis

The analytic sample was restricted to participants who completed the PTSD scale in at least two of the three post-Katrina surveys (n = 728; 77.3% of the original sample). All analyses were conducted in Stata 15.1 (Stata Corp., College Station, TX, USA). First, descriptive statistics were computed for the 728 participants in the analytic sample. Differences between the participants included in these analyses and the participants who were excluded because they completed fewer than two post-Katrina measures of Katrina-specific PTSD symptoms were assessed.

Next, the association between pre-Katrina trauma and PTSD trajectory was assessed with a three-step approach to examine relationships between predictor variables and trajectory group membership (e.g., the extent to which pre-Katrina trauma predicts trajectory group membership) [38]. Latent class growth analysis (LCGA) was used to identify trajectory groups based on patterns of PTSD symptoms reported at the post-Katrina data collection timepoints (Step 1). LCGA creates groups of individuals who have similar patterns of PTSD symptoms over time. Further analyses can then examine predictors of membership in each of these trajectory groups [39,40]. Models were run with 1 to 10 classes (trajectories) using the *traj* command in Stata [41]. In these models, Time 1 was anchored at 1, Time 2 at 4, and Time 3 at 12, representing the number of years post-disaster when each outcome measurement (PTSD score) was taken.

To identify the model that best fit the data, we compared the Akaike Information Criterion (AIC), Bayesian Information Criterion (BIC), and mean posterior probabilities [39,42]. For the AIC and BIC, lower values indicate better fit. For mean posterior probabilities, higher values indicate better fit; for a well-fitting model, the average posterior probability value should be >0.7 for each subgroup [42]. We also considered theoretical criteria, such as parsimony [39], clinical significance [43], and interpretability [44], when choosing the model that best represented the data.

Step 2 involved assigning individuals to their most likely trajectory group using the predicted posterior probabilities of belonging to each group. These classifications were then included as outcomes in multinomial logistic regression models where the probability of belonging to each trajectory group depended on predictor variables [45]. To assess relationships between predictors and trajectory membership (Step 3), we ran a series of models, beginning with the crude model that included only pre-Katrina traumatic experiences as a predictor of PTSD trajectory. Subsequent models added baseline sociodemographic characteristics (age, race, relationship status, and number of public benefits received), indicators of Katrina-related trauma and hardship (e.g., home damage), post-Katrina trauma exposure, as well as pre-Katrina social support and psychological distress [31,32], with the fully adjusted model including all of these covariates.

Postestimation using the *margins* command in Stata was used to calculate predicted probabilities of being in a given PTSD trajectory based on predictor variables. We estimated the predicted probability of being in a particular trajectory group for women with trauma counts equal to the sample mean value and at one standard deviation above the mean. We then calculated the difference between these two predicted probabilities to estimate the difference in the probability associated with a one standard deviation increase in trauma exposure. For example, women in the analytic sample reported an average of 1.66 pre-Katrina traumatic events, with a standard deviation of 1.98. We estimated the predicted probabilities of trajectory membership for women who experienced 1.66 pre-Katrina traumas and those who experienced 3.64 pre-Katrina traumas. The difference in the predicted probability of membership in a given trajectory for women who experienced 1.66 pre-Katrina traumas and women who experienced 3.64 pre-Katrina traumas represents the change in the predicted probability of trajectory membership associated with a one standard deviation increase in pre-Katrina trauma exposure.

To make changes in predicted probability more directly comparable across trajectories, we also calculated the relative change in probability. This was performed because the same change in predicted probability associated with greater trauma exposure may not be as meaningful for trajectories with a higher baseline predicted probability compared to trajectories with a lower baseline predicted probability. For example, a change in predicted probability of 0.05 is a larger relative change for a trajectory with a predicted probability of 0.10 (relative change of 50%) than for a trajectory with a predicted probability of 0.50 (relative change of 10%). Relative change was calculated by dividing the change in predicted probability associated with a one standard deviation increase in trauma exposure by the predicted probability of trajectory membership at the mean level of trauma exposure. Missing data on PTSD symptoms were estimated using maximum likelihood in the LCGA models. Missing data on predictors were imputed using chained multiple imputation.

## 3. Results

### 3.1. Preliminary Analyses

Table 1 shows descriptive statistics for the 728 women in the analytic sample. At baseline, the average participant’s age was 25.14 years (SD = 4.46) and 23.7% of participants were married or cohabitating with a partner. Most participants identified as non-Hispanic Black (86.1%). Using the validated K6 score cut points [36], before Hurricane Katrina, 17.9% of participants had probable mild to moderate mental illness and 5.9% had probable serious mental illness. Using the validated IES-R cut point [25], at Time 1 (one year post-Katrina), 48.1% of the sample met the criteria for probable Katrina-specific PTSD. At Time 2 (four years post-Katrina), 35.8% of participants had probable Katrina-specific PTSD, and at Time 3 (12 years post-Katrina), 18.7% had probable Katrina-specific PTSD.

Study participants experienced a high level of trauma exposure before, during, and after Hurricane Katrina. Overall, 61.3% of participants experienced at least one traumatic event prior to Hurricane Katrina, with a mean of 1.66 pre-Katrina traumatic experiences (SD = 1.98). Participants experienced an average of 0.80 (SD = 1.12) assaultive traumas and 0.87 (SD = 1.20) non-assaultive pre-Katrina traumas. Nearly 90% experienced at least one post-Katrina traumatic event (mean number of post-Katrina traumas = 2.60, SD = 1.99). On average, women reported experiencing 3.00 Katrina-related traumas on the eight-item hurricane trauma scale (SD = 2.29). Nearly 40% of participants experienced the death of a family member or friend due to Katrina and 83.4% had moderate to severe home damage as a result of Hurricane Katrina.

### 3.2. Latent Class Growth Analysis

Table 2 and Table 3 show the results of the latent class growth analysis models. Based on the model fit criteria shown in Table 2, the four-class model was chosen as the model that best represented the data. This model was selected because it had the lowest BIC value, an average posterior probability > 0.7, and because the trajectories in the four-class model were distinctive and clinically meaningful [40]. AIC was slightly lower for the five-class model (−2493.54), but the five-trajectory plot showed that two of the five trajectories were very similar in shape, with both characterized as having relatively low PTSD symptoms at Time 1 which decrease slightly over time. Therefore, we determined that these trajectories were not distinct from each other and did not differ in clinical significance. Although alignment with previous findings was not used to select the best fitting model, the trajectories identified by the four-class model roughly correspond to the four “prototypical” PTSD trajectories [11,12,13,14,15].

**Figure 1:**
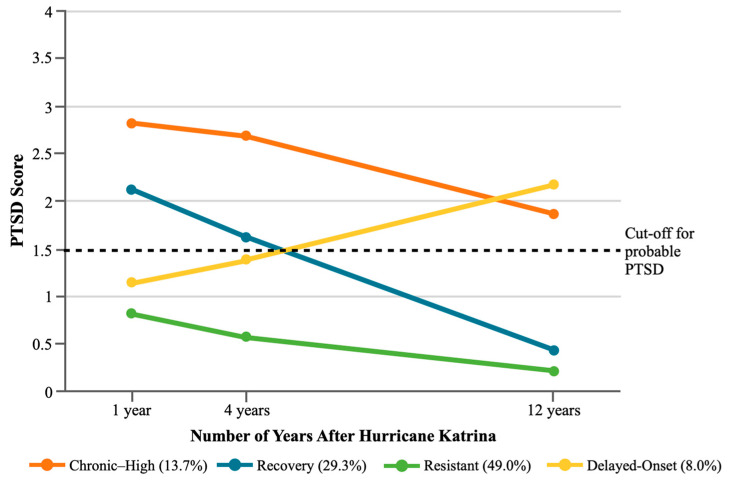
Post-traumatic stress disorder (PTSD) score trajectories from the four-class model.

The four final trajectories are shown in Figure 1. The “Resistant” trajectory, which encompasses 49.0% of participants, is characterized by low initial Katrina-specific PTSD symptoms that remain low over time. Nearly a third (29.3%) of participants were in a “Recovery” trajectory in which PTSD symptoms were initially high before decreasing over time. Approximately 8% of participants were in a “Delayed-Onset” trajectory characterized by PTSD symptoms that were initially low before increasing over time to cross the cut-off for probable PTSD. The final trajectory, “Chronic–High,” was the most severe of the four PTSD trajectories we identified. Participants in this trajectory (13.7%) experienced high PTSD symptoms at all three of the post-Katrina timepoints, with IES-R scores that surpassed the threshold for probable PTSD across the 12-year follow-up period. Table 4 shows descriptive statistics for the four subsamples with most likely membership in each trajectory.

### 3.3. Predictors of Trajectory Membership

**Figure 2:**
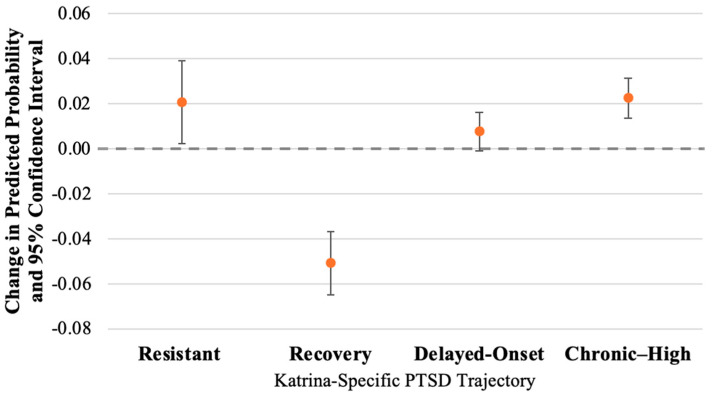
Change in predicted probability of PTSD trajectory group membership associated with a one SD increase in pre-Katrina trauma score.

Figure 2 shows the differences in the predicted probability of membership in each trajectory group associated with a one standard deviation (SD) increase in number of pre-Katrina traumatic events. Table 5 lists the predicted probabilities of membership in each trajectory group by level of pre-Katrina trauma exposure. In the unadjusted models, greater pre-Katrina trauma exposure was associated with an increased predicted probability of the Delayed-Onset and Chronic–High trajectories, and a decreased probability of membership in the Resistant and Recovery trajectories. For women with pre-Katrina trauma scores set at the mean (1.66 pre-Katrina traumas), the predicted probabilities of membership in the Delayed-Onset and Chronic–High trajectories were 0.08 (95% CI: 0.06, 0.10) and 0.13 (95% CI: 0.10, 0.15), respectively, in the unadjusted models. For women with pre-Katrina trauma scores one standard deviation above the mean (3.64 pre-Katrina traumas), the predicted probability increased to 0.10 (95% CI: 0.07, 0.13) for the Delayed-Onset trajectory and 0.18 (95% CI: 0.14, 0.22) for the Chronic–High trajectory, leading to relative increases of 21.3% and 43.0%, respectively. A one standard deviation increase in number of pre-Katrina traumas was associated with decreases in the predicted probability of trajectory group membership of 0.05 (95% CI: −0.07, −0.03) for the Resistant group and 0.02 (95% CI: −0.04, −0.01) for the Recovery group. These are relative decreases in probability of 10.1% for the Resistant trajectory and 7.4% for the Recovery trajectory.

These associations were attenuated for the Delayed-Onset and Chronic–High trajectories in the fully adjusted models. In the fully adjusted models, greater pre-Katrina trauma exposure was only associated with the Chronic–High and Recovery trajectories, although these associations were fairly small. After adjustment for covariates, a one standard deviation increase in number of pre-Katrina traumas was associated with a −17.2% relative decrease in the probability of being in the Recovery trajectory and a 17.3% relative increase in the predicted probability of Chronic–High PTSD. The probability of membership in the Recovery trajectory was 0.30 (95% CI: 0.26, 0.33) for women with the mean level of pre-Katrina trauma exposure and 0.24 (95% CI: 0.20, 0.29) for women with pre-Katrina trauma exposure that was one standard deviation above the mean level. A one standard deviation increase in number of pre-Katrina traumas was associated with an increase in the probability of Chronic–High PTSD from 0.13 (95% CI: 0.11, 0.15) to 0.15 (95% CI: 0.12, 0.19).

In the fully adjusted models, a one standard deviation increase in pre-Katrina trauma score was associated with a 9.7% relative increase in the predicted probability of Delayed-Onset PTSD. Finally, the direction of the association between greater pre-Katrina trauma exposure and the probability of the Resistant trajectory changed in the fully adjusted model, from a relative decrease of −10.1% in the crude model to a relative increase of 4.2% in the fully adjusted model. However, the changes in the probability of the Resistant and Delayed-Onset trajectories were small in magnitude and, therefore, may not represent meaningful effects.

The Delayed-Onset trajectory was the only trajectory for which there was a potentially meaningful difference between the extent to which assaultive and non-assaultive trauma predicted trajectory membership. For this trajectory, a one standard deviation increase in pre-Katrina assaultive traumatic events was associated with an increase in the predicted probability of 0.02 (95% CI = 0.01, 0.03) for Delayed-Onset PTSD, a relative increase of 22.2%. In contrast, a one standard deviation increase in non-assaultive pre-Katrina trauma was associated with a decrease in the predicted probability of 0.01 (95% CI = −0.01, −0.001), a relative decrease of −8.9%. This is consistent with prior research showing that assaultive traumas related to interpersonal violence, such as physical abuse or sexual assault, have a greater negative effect on mental health than non-assaultive traumas [6,27,46].

We assessed the change in the predicted probability of PTSD trajectory membership associated with a one standard deviation increase in number of pre-Katrina traumas after each covariate was added to the analytic model. The predicted probabilities of trajectory membership for each model can be found in Table S1 in the Supplementary Material. The magnitude of change in the predicted probability was relatively stable within trajectories as pre-Katrina sociodemographics, perceived social support, and psychological distress were added to the models. The association between greater pre-Katrina trauma exposure and Chronic–High Katrina-specific PTSD was attenuated when Katrina-related trauma was added to the model. In Model 4, which included pre-Katrina trauma, pre-Katrina sociodemographics, perceived social support, and psychological distress, the change in the probability of membership in the Chronic–High trajectory associated with a one standard deviation increase in pre-Katrina trauma exposure was 0.05 (95% CI: 0.03, 0.06). After the addition of the Katrina-related trauma scale score [33] and indicators of moderate to severe home damage and experiencing the death of a family member or close friend due to Katrina, the difference in the probability of Chronic–High PTSD associated with greater pre-Katrina trauma exposure was attenuated to 0.03 (95% CI: 0.02, 0.04).

The association between pre-Katrina trauma exposure and the probability of Delayed-Onset PTSD was attenuated most by the addition of post-Katrina trauma to the model (Model 8). The change in the predicted probability of the Resistant trajectory associated with higher pre-Katrina trauma exposure attenuated with the addition of each variable to the multinomial regression models, with the exception of post-Katrina trauma. In the model with pre-Katrina sociodemographics, perceived social support, psychological distress, and all three measures of Katrina-related trauma, pre-Katrina trauma had no impact on the probability of the Resistant trajectory. However, after the addition of post-Katrina trauma exposure, pre-Katrina trauma was associated with a small, positive change in the probability of the Resistant trajectory (0.02; 95% CI: 0.002, 0.04). In contrast, the association between the probability of the Recovery trajectory and pre-Katrina trauma exposure strengthened slightly with each additional variable added to the model.

Finally, because pre-Katrina trauma may indirectly impact Katrina-specific PTSD by increasing the severity of Katrina-related trauma, we also examined the extent to which Katrina-related traumas affected Katrina-specific PTSD trajectories. These results can be found in Table S2 in the Supplementary Material.

## 4. Discussion

The current study evaluated the extent to which pre-Katrina trauma exposure can explain differences in long-term trajectories of Katrina-specific PTSD among survivors with similar levels of Katrina-related trauma. First, four PTSD trajectories were identified. These trajectories roughly correspond to the four “prototypical” PTSD trajectories [11,12,13,14,15]. Nearly half (49.0%) of participants were in the Resistant trajectory, which is the least severe of the four trajectories and is characterized by PTSD symptoms that remained low over the 12 years of follow-up. Nearly a third of participants (29.3%) were in a trajectory of initially high Katrina-specific PTSD symptoms that decreased over time (Recovery). We identified two additional trajectories, both of which represent people who experience long-term PTSD symptoms. These are the Delayed-Onset trajectory (8.0%), in which PTSD symptoms were initially low after Hurricane Katrina, but subsequently increased, and the Chronic–High trajectory (13.7%), where people have high PTSD symptoms that persist over time.

In line with previous research, the most prevalent PTSD trajectory was the Resistant trajectory, with fewer participants following the Recovery, Delayed-Onset, and Chronic–High trajectories [47]. However, the proportion of women who were in the Resistant trajectory was lower than anticipated based on findings from previous studies. For example, a meta-analysis that examined the prevalence of PTSD trajectories in 35 studies [47] found that 69.5% of trauma survivors followed a trajectory similar to the Resistant trajectory, in which PTSD symptoms are low in the initial post-disaster period and remain low over time. The lower proportion of participants in our sample following this trajectory may be partially attributable to the fact that the majority of the participants identified as Black and all were female and low-income at baseline, all of which are characteristics associated with a higher risk of post-disaster mental health problems [4,11]. In addition, this study population is more highly traumatized than the majority of participants in studies included in the meta-analysis [47], which may reduce the probability of following a trajectory in which PTSD symptoms are low in both the short- and long-term aftermath of a disaster.

Our primary hypothesis was that experiencing greater pre-Katrina trauma would be associated with a higher risk of experiencing severe Katrina-specific PTSD, corresponding to the Chronic–High trajectory we found. We found that experiencing a greater number of traumatic events prior to Hurricane Katrina was associated with a higher probability of Chronic–High PTSD in the unadjusted model and in the model adjusting for pre-Katrina sociodemographics, perceived social support, and psychological distress. However, after adjusting for Katrina-related trauma and post-Katrina trauma, the association between pre-Katrina trauma exposure and the probability of Chronic–High Katrina-specific PTSD was attenuated. Higher pre-Katrina trauma exposure increased the probability of the Chronic–High trajectory slightly. Higher pre-Katrina trauma exposure moderately decreased the probability of being in the Recovery trajectory, in which Katrina-specific PTSD symptoms are initially high before declining over time. Pre-Katrina trauma did not have a significant effect on the probability of the Resistant and Delayed-Onset PTSD trajectories.

Of the various types of trauma exposure examined, Chronic–High PTSD was most strongly influenced by Katrina-related trauma. When covariates were added to the model one at a time, the association between pre-Katrina trauma and Chronic–High PTSD was attenuated most by the addition of Katrina-related trauma. This suggests that while pre-Katrina trauma may impact PTSD by increasing the severity of Katrina-related trauma, pre-Katrina trauma exposure does not have a strong direct effect on chronic Katrina-specific PTSD. Of the three types of Katrina-related trauma examined, the probability of Chronic–High PTSD was most strongly associated with experiencing the death of a family member or close friend due to Katrina and moderate to severe home damage (see Supplemental Material for full results). The coding of the home damage variable encompassed a relatively wide degree of damage; it is possible that an even stronger relationship with Chronic–High PTSD may be observed if home damage was restricted to the most severe category of damage only.

In line with the conservation of resources theory, pre-disaster trauma may lead to losses of social, economic, and psychological resources, creating conditions for greater severity of future traumatic events [48]. For example, individuals who have experienced a loss of resources due to a pre-disaster traumatic event may have more difficulty preparing for a disaster, or evacuating from their home. Specifically, having greater social resources can facilitate evacuation plans and reduce barriers to evacuation by enabling individuals to more easily find help with transportation or housing during evacuation [23,49]. Similarly, having more material resources, such as owning a car or having a higher income, can reduce barriers to evacuation and limit exposure to disaster-related trauma [23]. Inability to evacuate may then lead to greater exposure to disaster-related traumatic events, such as lacking food, fresh water, or medical care in the aftermath of the disaster, or witnessing a friend or family member be seriously injured or die as a result of the disaster [23]. Because the degree of disaster-related traumatic events strongly predicts post-disaster PTSD, pre-Katrina trauma may impact Katrina-specific PTSD through the severity of Katrina-related traumatic events [4].

## 5. Limitations and Conclusions

This study has several limitations. First, PTSD symptoms were assessed using a self-reported measure, which may be less accurate than a clinical assessment and cannot provide a definitive diagnosis of PTSD. Second, there is a significant time lag between the second and third post-Katrina PTSD assessments, and the number of years between surveys is not consistent. It is possible that the trajectories we identified in these analyses may be different from the trajectories we may have identified if we had access to additional waves of data collected between the second and third post-Katrina surveys. Third, although we examined the extent to which assaultive vs. non-assaultive traumas had differing effects on Katrina-specific PTSD and found little difference between the effects of these types of trauma, we were not able to assess which specific traumatic events are most associated with post-disaster PTSD trajectory due to sample size. Future research should further parse out the types of trauma that confer the greatest risk of chronic post-disaster PTSD. Fourth, because pre- and post-Katrina trauma were retrospectively reported, they may be affected by recall bias. Finally, although this sample is representative of the population that is most vulnerable to adverse post-disaster mental health outcomes [4,11], it is not representative of all Hurricane Katrina survivors and these findings may not be generalizable to survivors of other disasters.

Despite these limitations, this study provides key insight into the course of disaster recovery among the most vulnerable disaster survivors, including people with a high burden of trauma. The results from this study suggest that while pre-disaster trauma exposure does not have a strong direct effect on chronic disaster-specific PTSD, pre-disaster trauma may impact PTSD through other factors that affect disaster-related PTSD, such as by increasing the severity of disaster-related trauma. These findings have important implications for the development of disaster preparedness and response strategies to diminish the long-term burden of disaster-related PTSD. Because pre-disaster resource loss may create conditions for greater severity of disaster-related traumatic events [27,28], interventions that help to rebuild these resources could reduce future disaster-related trauma exposure. Applying these findings to the development of disaster response strategies and interventions has the potential to prevent the most vulnerable disaster survivors from experiencing years of disaster-related PTSD.

## Figures and Tables

**Table 1:** Descriptive statistics for analytic sample (n = 728).

		Mean (SD) or %
Baseline sociodemographics		
	Age (years)	25.14 (4.46)
	Non-Hispanic Black	86.1%
	Married or cohabitating	23.7%
	Number of public benefits received (range: 0–4)	0.94 (0.71)
Pre-Katrina perceived social support (range: 1–4)		3.20 (0.44)
Pre-Katrina psychological distress (range: 0–24)		4.97 (4.18)
Pre-Katrina trauma exposure		
	Experienced any pre-Katrina trauma	61.3%
	Number of pre-Katrina traumas experienced (range: 0–14)	1.66 (1.98)
	Number of pre-Katrina assaultive traumas experienced (range: 0–5)	0.80 (1.12)
	Number of pre-Katrina non-assaultive traumas experienced (range: 0–9)	0.87 (1.20)
Katrina-related trauma and hardship		
	Number of Katrina-related traumas (range: 0–8)	3.00 (2.29)
	Family member or friend died due to Katrina	39.3%
	Moderate or severe home damage due to Katrina	83.4%
Post-Katrina trauma exposure		
	Experienced any post-Katrina trauma	87.4%
	Number of post-Katrina traumas experienced (range: 0–15)	2.60 (1.99)
Katrina-specific post-traumatic stress disorder ^1^ (PTSD)		
	Time 1 (one year post-Katrina)	48.1%
	Time 2 (four years post-Katrina)	35.8%
	Time 3 (12 years post-Katrina)	18.7%

^1^ Probable Katrina-specific PTSD is indicated by an IES-R score > 1.5 [23].

**Table 2:** Fit statistics for the latent class growth analysis models (model selected as best representation of the data is bolded).

Number of Classes	AIC	BIC	Adj. BIC	Mean Posterior Probability (SD)
1	−2662.18	−2669.07	−2670.51	--
2	−2528.34	−2542.11	−2545.00	0.91 (0.04)
3	−2516.81	−2537.46	−2541.79	0.80 (0.10)
**4**	**−2494.79**	**−2522.34**	**−2528.11**	**0.79 (0.06)**
5	−2493.54	−2527.97	−2535.18	0.70 (0.08)
6	−2496.54	−2537.85	−2546.51	0.51 (0.29)
7	−2499.54	−2547.74	−2557.84	0.35 (0.30)
8	−2502.54	−2557.62	−2569.17	0.32 (0.31)
9	−2505.54	−2567.51	−2580.49	0.27 (0.29)
10	−2504.06	−2572.92	−2587.35	0.30 (0.33)

**Table 3:** Percentage of participants in each class.

Number of Classes	1	2	3	4	5	6	7	8	9	10
1	100.0%									
2	71.8%	28.2%								
3	59.8%	22.3%	17.9%							
4	49.0%	8.0%	29.3%	13.7%						
5	32.5%	33.3%	7.8%	13.3%	13.2%					
6	15.5%	7.8%	17.0%	33.3%	13.3%	13.2%				
7	15.4%	7.8%	17.1%	16.1%	17.2%	13.3%	13.2%			
8	12.5%	7.8%	10.8%	9.2%	15.4%	17.8%	13.3%	13.2%		
9	11.5%	7.8%	11.7%	9.3%	10.9%	11.7%	10.6%	13.3%	13.2%	
10	8.6%	9.6%	8.0%	13.7%	10.7%	12.2%	9.5%	12.6%	13.8%	1.3%

**Table 4:** Descriptive statistics for participants with most likely membership in each PTSD symptom trajectory.

		Trajectory Group			
		Resistant (n = 357, 49.0%)	Recovery (n = 213, 29.3%)	Delayed-Onset (n = 58, 8.0%)	Chronic–High (n = 100, 13.7%)
		Mean (SD) or %	Mean (SD) or %	Mean (SD) or %	Mean (SD) or %
Baseline sociodemographics					
	Age (years)	24.58 (4.18)	25.54 (4.70)	26.03 (5.05)	26.35 (4.24)
	Non-Hispanic Black	80.2%	91.0%	92.7%	92.8%
	Married or cohabitating	26.3%	20.2%	22.8%	22.2%
	Number of public benefits received (range: 0–4)	0.86 (0.74)	0.99 (0.70)	0.97 (0.59)	1.09 (0.70)
Pre-Katrina perceived social support (range: 1–4)		3.27 (0.42)	3.15 (0.47)	3.17 (0.41)	3.09 (0.45)
Pre-Katrina psychological distress (range: 0–24)		4.31 (3.70)	5.53 (4.34)	5.32 (4.62)	5.92 (4.82)
Pre-Katrina trauma exposure					
	Experienced any pre-Katrina trauma	58.2%	59.6%	70.2%	69.2%
	Number of pre-Katrina traumas experienced (range: 0–14)	1.45 (1.82)	1.47 (1.61)	2.04 (2.05)	2.52 (2.75)
	Number of pre-Katrina assaultive traumas experienced (range: 0–5)	0.67 (0.99)	0.71 (1.01)	1.09 (1.24)	1.18 (1.50)
	Number of pre-Katrina non-assaultive traumas experienced (range: 0–9)	0.77 (1.15)	0.77 (1.01)	0.95 (1.12)	1.33 (1.59)
Katrina-related trauma and hardship					
	Number of Katrina-related traumas (range: 0–8)	2.27 (1.93)	3.41 (2.32)	3.46 (2.29)	4.46 (2.44)
	Family member or friend died due to Katrina	26.1%	50.2%	35.1%	65.0%
	Moderate or severe home damage due to Katrina	78.3%	89.1%	77.4%	92.7%
Post-Katrina trauma exposure					
	Experienced any post-Katrina trauma	86.2%	86.9%	93.0%	89.0%
	Number of post-Katrina traumas experienced (range: 0–15)	2.19 (1.66)	2.66 (1.94)	3.28 (2.10)	3.45 (2.55)
Katrina-specific post-traumatic stress disorder (PTSD)					
	Time 1 (one year post-Katrina)	0.82 (0.59)	2.12 (0.64)	1.14 (0.59)	2.82 (0.53)
	Time 2 (four years post-Katrina)	0.57 (0.52)	1.61 (0.68)	1.39 (0.76)	2.69 (0.62)
	Time 3 (twelve years post-Katrina)	0.21 (0.34)	0.43 (0.46)	2.17 (0.60)	1.87 (0.76)

**Table 5:** Predicted probability and 95% confidence intervals of PTSD trajectory membership by level of pre-Katrina trauma exposure.

			Trajectory Group							
			Resistant		Recovery		Delayed-Onset		Chronic–High	
			Crude	Fully Adjusted	Crude	Fully Adjusted	Crude	Fully Adjusted	Crude	Fully Adjusted
**Pre-Katrina Trauma Exposure ^1^**										
	Predicted probability at mean level of pre-Katrina trauma exposure		0.50 (0.46, 0.53)	0.50 (0.46, 0.53)	0.30 (0.26, 0.33)	0.30 (0.26, 0.33)	0.08 (0.06, 0.10)	0.08 (0.06, 0.10)	0.13 (0.10, 0.15)	0.13 (0.11, 0.15)
	Predicted probability at 1 SD above mean		0.45 (0.39, 0.50)	0.52 (0.46, 0.57)	0.27 (0.22, 0.32)	0.24 (0.20, 0.29)	0.10 (0.07, 0.13)	0.09 (0.06, 0.12)	0.18 (0.14, 0.22)	0.15 (0.12, 0.19)
	*Difference*		−0.05 (−0.07, −0.03)	0.02 (0.002, 0.04)	−0.02 (−0.04, −0.01)	−0.05 (−0.06, −0.04)	0.02 (0.01, 0.03)	0.01 (−0.001, 0.02)	0.05 (0.04, 0.07)	0.02 (0.01, 0.03)
	*Percent change in probability*		−10.1%	4.2%	−7.4%	−17.2%	21.3%	9.7%	43.0%	17.3%
	**Pre-Katrina Assaultive Trauma Exposure**									
		Predicted probability at mean level of pre-Katrina assaultive trauma exposure	0.50 (0.46, 0.53)	0.49 (0.46, 0.53)	0.30 (0.26, 0.33)	0.29 (0.26, 0.33)	0.08 (0.06, 0.10)	0.08 (0.06, 0.10)	0.13 (0.10, 0.15)	0.13 (0.11, 0.16)
		Predicted probability at 1 SD above mean	0.45 (0.39, 0.50)	0.49 (0.43, 0.55)	0.28 (0.23, 0.33)	0.27 (0.22, 0.32)	0.10 (0.07, 0.13)	0.10 (0.06, 0.13)	0.18 (0.14, 0.21)	0.14 (0.11, 0.18)
		*Difference*	−0.05 (−0.07, −0.03)	−0.003 (−0.03, 0.02)	−0.02 (−0.03, −0.002)	−0.02 (−0.04, −0.01)	0.02 (0.01, 0.03)	0.02 (0.01, 0.03)	0.05 (0.03, 0.06)	0.01 (0.001, 0.02)
		*Percent change in probability*	−10.1%	−0.6%	−5.7%	−8.3%	26.4%	22.2%	35.5%	7.5%
	**Pre-Katrina Non-Assaultive Trauma Exposure**									
		Predicted probability at mean level of pre-Katrina non-assaultive trauma exposure	0.50 (0.46, 0.53)	0.49 (0.46, 0.53)	0.29 (0.26, 0.33)	0.29 (0.26, 0.33)	0.08 (0.06, 0.10)	0.08 (0.06, 0.10)	0.13 (0.10, 0.15)	0.13 (0.11, 0.16)
		Predicted probability at 1 SD above mean	0.46 (0.41, 0.52)	0.52 (0.47, 0.57)	0.27 (0.22, 0.33)	0.26 (0.21, 0.31)	0.09 (0.06, 0.12)	0.07 (0.05, 0.10)	0.18 (0.14, 0.21)	0.15 (0.11, 0.18)
		*Difference*	−0.04 (−0.05, −0.02)	0.03 (0.01, 0.05)	−0.02 (−0.04, −0.002)	−0.04 (−0.05, −0.02)	0.01 (−0.002, 0.02)	−0.01 (−0.01, −0.001)	0.05 (0.03, 0.06)	0.02 (0.01, 0.02)
		*Percent change in probability*	−7.1%	5.7%	−6.6%	−12.5%	9.5%	−8.9%	36.5%	12.2%

^1^ Adjusted model includes baseline age, race, partnership status, and number of public benefits received, pre-Katrina psychological distress and perceived social support, and Katrina-related and post-Katrina trauma exposure. Models for pre-Katrina assaultive trauma also control for pre-Katrina non-assaultive trauma, and vice versa.

## Data Availability

The data presented in this study are available on request from the corresponding author.

## Supplementary Materials

The following supporting information can be downloaded at: https://www.mdpi.com/article/10.3390/ijerph21060749/s1, Table S1: Results of multinomial regression models estimating predicted probability of trajectory group membership; Table S2: Predicted probability and 95% confidence intervals of PTSD trajectory membership by level and type of Katrina-related trauma exposure.
