# Supplementary material for: Predicting Post-Disaster Post-Traumatic Stress Disorder Symptom Trajectories: The Role of Pre-Disaster Traumatic Experiences
Source: Int J Environ Res Public Health. 2024 Jun 8;21(6):749. doi: 10.3390/ijerph21060749 (PMC11204121; doi:10.3390/ijerph21060749)
Supplement: Supplementary file 1 [file ijerph-21-00749-s001.zip › ijerph-3014871-supplementary.pdf]

Supplemental Material for “Predicting Post-Disaster Post-Traumatic Stress Disorder Symptom Trajectories: The Role of Pre-Disaster Traumatic Experiences”

Table S1. Results of multinomial regression models estimating predicted probability of trajectory group membership

		Trajectory Group			
**Model #**	**Model Covariates**	**Resistant**	**Recovery**	**Delayed-Onset**	**Chronic-High**
1 (Crude)	Pre-Katrina trauma only				
	Predicted probability at mean level of pre-Katrina trauma exposure	0.50 (0.46, 0.53)	0.30 (0.26, 0.33)	0.08 (0.06, 0.10)	0.13 (0.10, 0.15)
	Predicted probability at 1 SD above mean	0.45 (0.39, 0.50)	0.27 (0.22, 0.32)	0.10 (0.07, 0.13)	0.18 (0.14, 0.22)
	Difference	-0.05 (-0.07, -0.03)	-0.02 (-0.04, -0.01)	0.02 (0.01, 0.03)	0.05 (0.04, 0.07)
	Percent change in probability	-10.1%	-7.4%	21.3%	43.0%
2	Model 1 + pre-Katrina sociodemographics				
	Predicted probability at mean level of pre-Katrina trauma exposure	0.50 (0.46, 0.53)	0.30 (0.26, 0.33)	0.08 (0.06, 0.10)	0.13 (0.10, 0.15)
	Predicted probability at 1 SD above mean	0.45 (0.40, 0.50)	0.27 (0.22, 0.32)	0.10 (0.07, 0.13)	0.18 (0.14, 0.22)
	Difference	-0.05 (-0.06, -0.03)	-0.02 (-0.04, -0.01)	0.02 (0.01, 0.03)	0.05 (0.04, 0.06)
	Percent change in probability	-9.1%	-8.3%	23.4%	39.9%
3	Model 2 + pre-Katrina social support				
	Predicted probability at mean level of pre-Katrina trauma exposure	0.50 (0.46, 0.53)	0.30 (0.26, 0.33)	0.08 (0.06, 0.10)	0.13 (0.10, 0.15)
	Predicted probability at 1 SD above mean	0.46 (0.40, 0.51)	0.27 (0.22, 0.32)	0.10 (0.07, 0.13)	0.18 (0.14, 0.21)
	Difference	-0.04 (-0.06, -0.02)	-0.03 (-0.04, -0.01)	0.02 (0.01, 0.03)	0.05 (0.04, 0.06)
	Percent change in probability	-8.0%	-9.1%	22.9%	37.7%
4	Model 3 + pre-Katrina psychological distress				
	Predicted probability at mean level of pre-Katrina trauma exposure	0.50 (0.46, 0.53)	0.30 (0.26, 0.33)	0.08 (0.06, 0.10)	0.13 (0.10, 0.15)
	Predicted probability at 1 SD above mean	0.46 (0.41, 0.51)	0.26 (0.22, 0.31)	0.10 (0.07, 0.13)	0.17 (0.14, 0.21)
	Difference	-0.03 (-0.05, -0.02)	-0.03 (-0.05, -0.02)	0.02 (0.01, 0.03)	0.05 (0.03, 0.06)
	Percent change in probability	-6.7%	-10.8%	23.6%	36.0%
5	Model 4 + Katrina-related trauma scale				
	Predicted probability at mean level of pre-Katrina trauma exposure	0.49 (0.46, 0.53)	0.30 (0.26, 0.33)	0.08 (0.06, 0.10)	0.13 (0.10, 0.15)
	Predicted probability at 1 SD above mean	0.48 (0.43, 0.53)	0.26 (0.21, 0.31)	0.10 (0.07, 0.13)	0.16 (0.13, 0.20)

	Difference	-0.01 (-0.03, 0.01)	-0.04 (-0.05, -0.02)	0.02 (0.01, 0.03)	0.03 (0.02, 0.04)
	Percent change in probability	-2.3%	-13.2%	20.0%	26.8%
6	Model 5 + Katrina-related home damage				
	Predicted probability at mean level of pre-Katrina trauma exposure	0.49 (0.46, 0.53)	0.30 (0.26, 0.33)	0.08 (0.06, 0.10)	0.13 (0.10, 0.15)
	Predicted probability at 1 SD above mean	0.49 (0.44, 0.54)	0.25 (0.21, 0.30)	0.10 (0.07, 0.13)	0.16 (0.13, 0.20)
	Difference	-0.01 (-0.02, 0.01)	-0.04 (-0.06, -0.03)	0.02 (0.01, 0.03)	0.03 (0.02, 0.04)
	Percent change in probability	-1.7%	-14.2%	22.5%	25.2%
7	Model 6 + Katrina-related bereavement				
	Predicted probability at mean level of pre-Katrina trauma exposure	0.49 (0.46, 0.53)	0.30 (0.26, 0.33)	0.08 (0.06, 0.10)	0.13 (0.10, 0.15)
	Predicted probability at 1 SD above mean	0.49 (0.44, 0.54)	0.25 (0.21, 0.30)	0.10 (0.07, 0.13)	0.16 (0.13, 0.19)
	Difference	-0.004 (-0.02, 0.01)	-0.04 (-0.06, -0.03)	0.02 (0.01, 0.03)	0.03 (0.02, 0.04)
	Percent change in probability	-0.84%	-15.1%	22.9%	23.7%
8 (Fully-Adjusted)	Model 7 + post-Katrina trauma				
	Predicted probability at mean level of pre-Katrina trauma exposure	0.50 (0.46, 0.53)	0.30 (0.26, 0.33)	0.08 (0.06, 0.10)	0.13 (0.11, 0.15)
	Predicted probability at 1 SD above mean	0.52 (0.46, 0.57)	0.24 (0.20, 0.29)	0.09 (0.06, 0.12)	0.15 (0.12, 0.19)
	Difference	0.02 (0.002, 0.04)	-0.05 (-0.06, -0.04)	0.01 (-0.001, 0.02)	0.02 (0.01, 0.03)
	Percent change in probability	4.2%	-17.2%	9.7%	17.3%

Table S2. Predicted probability and 95% confidence intervals of PTSD trajectory membership by level and type of Katrina-related trauma exposure

	Trajectory Group							
	**Resistant**		**Recovery**		**Delayed-Onset**		**Chronic-High**	
	Crude	Fully Adjusted	Crude	Fully Adjusted	Crude	Fully Adjusted	Crude	Fully Adjusted
**Katrina-Related Trauma Exposure**[1]								
Predicted probability at mean level of Katrina-related trauma exposure	0.49 (0.45, 0.53)	0.49 (0.45, 0.52)	0.31 (0.27, 0.34)	0.31 (0.27, 0.34)	0.08 (0.06, 0.10)	0.08 (0.06, 0.10)	0.12 (0.10, 0.15)	0.12 (0.10, 0.15)
Predicted probability at 1 SD above mean	0.32 (0.27, 0.38)	0.39 (0.33, 0.44)	0.36 (0.31, 0.41)	0.34 (0.28, 0.39)	0.10 (0.07, 0.13)	0.10 (0.07, 0.14)	0.22 (0.18, 0.26)	0.18 (0.14, 0.21)
Difference	-0.17 (-0.18, -0.15)	-0.10 (-0.12, -0.08)	0.06 (0.04, 0.07)	0.03 (0.01, 0.05)	0.01 (0.01, 0.03)	0.02 (0.01, 0.03)	0.10 (0.08, 0.11)	0.05 (0.04, 0.06)
Percent change in probability	-34.0%	-21.0%	17.9%	10.3%	17.9%	23.1%	78.4%	41.7%
**Moderate to Severe Home Damage Due to Katrina**[1]								
Predicted probability – Did not experience moderate to severe home damage	0.64 (0.55, 0.73)	0.56 (0.47, 0.65)	0.19 (0.12, 0.27)	0.22 (0.14, 0.31)	0.11 (0.05, 0.17)	0.13 (0.06, 0.20)	0.06 (0.02, 0.10)	0.08 (0.02, 0.14)
Predicted probability – Experienced moderate to severe home damage	0.46 (0.42, 0.50)	0.48 (0.44, 0.51)	0.31 (0.27, 0.35)	0.31 (0.27, 0.34)	0.07 (0.05, 0.09)	0.07 (0.05, 0.09)	0.15 (0.12, 0.18)	0.14 (0.12, 0.17)
Difference	-0.18 (-0.13, -0.23)	-0.09 (-0.14, -0.03)	0.12 (0.08, 0.16)	0.09 (0.04, 0.13)	-0.04 (-0.07, 0.001)	-0.06 (-0.11, -0.01)	0.10 (0.08, 0.11)	0.06 (0.03, 0.09)
Percent change in probability	-27.9%	-15.4%	60.8%	38.7%	-32.7%	-45.5%	166.1%	74.8%
**Family Member or Close Friend Died Due to Katrina**[1]								
Predicted probability – Did not lose a family member or close friend due to Katrina	0.60 (0.55, 0.64)	0.55 (0.51, 0.59)	0.24 (0.20, 0.28)	0.26 (0.21, 0.30)	0.09 (0.06, 0.11)	0.09 (0.06, 0.12)	0.08 (0.05, 0.10)	0.10 (0.07, 0.13)
Predicted probability – Lost a family member or close friend due to Katrina	0.33 (0.27, 0.38)	0.40 (0.34, 0.45)	0.37 (0.32, 0.43)	0.36 (0.30, 0.42)	0.07 (0.04, 0.10)	0.07 (0.04, 0.10)	0.23 (0.18, 0.28)	0.18 (0.14, 0.22)
Difference	-0.27 (-0.28, -0.26)	-0.15 (-0.17, -0.14)	0.13 (0.12, 0.15)	0.10 (0.09, 0.12)	-0.01 (-0.02, -0.01)	-0.02 (-0.03, -0.02)	0.15 (0.12, 0.17)	0.08 (0.07, 0.09)
Percent change in probability	-45.1%	-28.1%	56.1%	39.3%	-16.1%	-26.7%	187.2%	78.3%

[1]Fully adjusted model includes baseline age, race, partnership status and number of public benefits received, pre-Katrina psychological distress and perceived social support, and pre-Katrina and post-Katrina trauma exposure.
